# New Method of Nickel-Plating

**Published:** 1872-12

**Authors:** 


### ARTICLE XIII.

### *New Method of Nickel-Plating.*

Within the last few years a process of nickel- plating by electrolysis has been invented by Mr. Isaac Adams, of Boston, and is now employed to a considerable extent in all of our large cities. Mr. Adams' process, which is patented, involves the employment of a bath of double sulphate of nickel and ammonium and of an anode of cast nickel. The prices charged by companies working under the patent are extremely exorbitant, and, as a coating of nickel is an excellent preventive against rust and injury by acid vapor, chemists will give a hearty welcome to a simple and cheap method of nickel-plating, which is open to the use of all. The process, which is due to Prof. F. Stolba, is as follows:— The salt of nickel may be the chloride, sulphate or double sulphate of nickel and potassium. It need not be chemically pure, but must contain no metals which are precipitated by zinc. In addition, the operator will require a solution of chloride of zinc, obtained by dissolving commercial zinc in common chlorhydric acid, cuttings of sheet zinc, zinc-dust and pure chlorhydric acid. The process of plating may be effected in a vessel of porcelain of metal; the author prefers copper, which itself becomes plated with nickel. The articles to be plated may be of cast or wrought iron, steel, copper, brass, zinc or lead. They must be completely immersed in the liquid used for plating, and their surfaces must be perfectly free from fat and rust. Iron vessels may be cleaned by treating with a solution containing 3 or 4 per cent. of chlorhydric acid. A sufficient quantity of concentrated solution of chloride of zinc is now poured into the plating vessel, and from once to twice its volume of water added. The soluiion is then to be heated to the boiling point, and chlorhydric acid added drop by drop until the precipitate, formed by diluting the chloride of zinc with water, is redissolved. As much zinc-powder as will cover the point of a knife is then added, by which the metal of the vessel becomes, in a few minutes, plated with zinc wherever it is in contact with this liquid. Enough nickel salt is then to be introduced to color the liquid distinctly green, after which the articles to be plated, and with them some small cuttings of zinc, are to be put in, care being taken to afford a sufficient number of points of contact. The liquid is then to be boiled, when the nickel is soon precipitated, and the work is finished in about

15 minutes. If particular parts of the articles are not plated, the boiling must be continued, fresh pieces of zinc, and, if necessary, fresh nickel salt, being added. It is important, if the coating of nickel is to be brilliant, that the liquid on boiling shall not be cloudy from basic zinc salt, or acid from free hydrochloric acid. The nickel-plated articles must be well washed with water and then cleaned with polishing chalk. The author found that articles of copper, plated with nickel, after several months' exposure to the atmosphere of the laboratory, appeared scarcely tarnished. It is important to remark that the same liquid may be employed repeatedly for nickel-plating, especially when chloride of nickel is employed. The same process applies to cobalt, but the coating with this metal, besides its cost, possesses no practical value.—*Polytechnisches Journal.*

